# Optimization and Characterization of Chitosan Enzymolysis by Pepsin

**DOI:** 10.3390/bioengineering3030017

**Published:** 2016-07-01

**Authors:** Bi Foua Claude Alain Gohi, Hong-Yan Zeng, A Dan Pan

**Affiliations:** Biotechnology Institute, College of Chemical Engineering, Xiangtan University, Xiangtan 411105, Hunan, China; claudefouabi@hotmail.fr (B.F.C.A.G.); jessciapan@hotmail.com (A.D.P.)

**Keywords:** chitosan, enzymolysis, pepsin, response surface methodology, inhibition, kinetic

## Abstract

Pepsin was used to effectively degrade chitosan in order to make it more useful in biotechnological applications. The optimal conditions of enzymolysis were investigated on the basis of the response surface methodology (RSM). The structure of the degraded product was characterized by degree of depolymerization (DD), viscosity, molecular weight, FTIR, UV-VIS, SEM and polydispersity index analyses. The mechanism of chitosan degradation was correlated with cleavage of the glycosidic bond, whereby the chain of chitosan macromolecules was broken into smaller units, resulting in decreasing viscosity. The enzymolysis by pepsin was therefore a potentially applicable technique for the production of low molecular chitosan. Additionally, the substrate degradation kinetics of chitosan were also studied over a range of initial chitosan concentrations (3.0~18.0 g/L) in order to study the characteristics of chitosan degradation. The dependence of the rate of chitosan degradation on the concentration of the chitosan can be described by Haldane’s model. In this model, the initial chitosan concentration above which the pepsin undergoes inhibition is inferred theoretically to be about 10.5 g/L.

## 1. Introduction

Chitosan is a natural polysaccharide, which is widely distributed among living organisms in nature and has been studied extensively in the last few decades. The promising utilization of chitosan in various fields, including medicine, pharmacology and the food industry, is due to the combination of its excellent biological properties, its biocompatibility, biodegradability and low toxicity. However, its limited application in medicine and the food industry is attributed to its high molecular weight, giving it low solubility in aqueous media. These limitations can be overcome by the hydrolysis of chitosan leading to the production of low molecular weight (LMW) chitosan (oligosaccharides) [1,2,3]. Hydrolysis of chitosan involves physical, chemical and enzymatic dissociation. Compared to the physical and chemical methods, enzymatic degradation has some advantages of specificity, mildness, easy control, no wastewater and easy separation of reactants. Enzymolysis can improve the functional properties of chitosan without affecting either its glucose ring, or its biological activity, while producing high quantities of chitooligosaccharides [1,4,5,6]. The degree of hydrolysis (DH) is an important parameter for determining the functional properties of LMW chitosan. The LMW chitosan with a free-amine group possesses high solubility in acid-free water and low viscosity. Water-solubility enables efficient modification for medical and agricultural applications [7]. LMW chitosan with a free amine was prepared by enzymolysis. It had an average molecular weight of 18,579 Da and a degree of depolymerization (DD) of 93% [8]. With free amine groups, the LMW chitosan has great solubility, which makes it a good candidate for DNA and drug delivery systems [9,10]. It also shows potential germicidal activities against pathogenic bacteria, yeast and filamentous fungus [11]. Many enzymes with different original specificities, such as cellulase, pectinase, pepsin, papain and lipase, have been reported to have the ability to hydrolyze chitosan [1]. Among these enzymes, pepsin has attracted the most attention from researchers on account of its capability to produce the highest yield of LMW chitosan in enzymolysis [12]. In spite of the merits of the proposed approach, the enzymolysis process of degrading chitosan by pepsin is not fully described in detail in the literature. The enzymolysis process of chitosan degradation by papain however has been exhaustively described in our previous study (Pan et al., 2016) [13].

The aim of this study was to understand and improve the enzymolysis process of degrading chitosan into the LMW chitosan. In order to investigate the relationships between the reaction variables (reaction temperature, initial pH, chitosan and pepsin concentrations) and the response (the degree of hydrolysis (DH)), the optimum enzymolysis condition was achieved using the statistical experimental design called the response surface methodology (RSM) analysis. Furthermore, in order to understand the reaction process and to choose the most suitable technology for chitosan enzymolysis, it was necessary to investigate the enzymolysis kinetics. Based on the results of this investigation, a comparative study will be established between our previous study [13] and this work. It would be useful to provide a scientific approach to a theoretic basis for chitosan enzymolysis with high performance and low consumption.

## 2. Materials and Methods

### 2.1. Materials

Chitosan from shrimp shells (≥91% deacetylated) and pepsin (EC 3.4.23.1, 3000–3500 units/mg protein) from porcine gastric mucosa (Amresco type A) were purchased from Sinopharm Chemical Reagent Co., Ltd. (Shanghai, China). All other reagents used were of analytical grade and they were used without further purification. All solutions were made with redistilled and ion-free water.

### 2.2. Chitosan Hydrolysis

Stock solution of pepsin (1.0 g/L) was obtained by dissolving the enzyme in Tris-HCl buffer (0.1 mol/L, pH 7.0). Stock solution of chitosan (10.0 g/L) was prepared in 0.2 mol/L acetic acid/sodium acetate (1:1, v/v) buffer, pH 4, and diluted into different concentrations for the assays of chitosan hydrolysis. In the preliminary experiment, the standard assay contained 1% w/v. Chitosan (≥91% deacetylated) was dissolved in 100 mM sodium acetate buffer at pH 4, and pepsin was dissolved in an enzyme/substrate ratio of 1:100 (w/w) at 50 °C. It was found that the reaction attained equilibrium after 70 min, so the reaction time for the hydrolysis was set to 70 min. The pepsin solutions were initially added to the buffers at different chitosan concentrations, respectively. The mixed solution was then maintained in a thermostatic water bath at a specific temperature and pH while stirring at 500 rpm for 70 min and heated to 95 °C for 10 min to terminate the reaction. After the reaction, the mixture was cooled to room temperature then centrifuged at 800 rpm for 5 min to remove the enzyme. The supernatant was stored to determine reducing sugars (SRSs) using a total organic carbon analyzer (TOC-5000A, Shimadzu, Japan). The tests were made in triplicate, and the results were recorded as an average. The SRS yield was calculated as follows:

SRSs yield (%) = (carbon mass of SRSs)/(carbon mass of chitosan) × 100%

The response surface methodology (RSM) represents a statistical method that uses quantitative data from an appropriate experimental design to determine and simultaneously solve multivariate equations. The main advantage of RSM is the reduced number of experimental trials needed to evaluate multiple parameters and their interactions.

After approximation of the best conditions by the “one-factor-at-a-time” method in our preliminary experiments, RSM was used to test the effect of initial pH, reaction temperature, pepsin concentration and chitosan concentration on the SRS yield in the chitosan enzymolysis. the Box–Behnken design (BBD) was chosen for the experiment with four independent variables of initial pH (*P*), reaction temperature (*T*, °C), pepsin concentration (*E*, g/L) and chitosan concentration (*C*, g/L), while optimizing one response variable, SRS yield (*Y*), from the enzymolysis process. Each independent variable was coded at three levels between −1 and +1, while the variables *p*, *T*, *E* and *C* were changed in the ranges shown in Table 1. A set of 29 experiments was augmented with three replications at the design center to evaluate the pure error. The experiments were carried out in a randomized order as required in many design procedures. After reaction, the response *Y* was measured, and the statistical software package Design Expert (Version 8.0.6) was used for regression analysis of the experimental data and to plot the response surface. Conducting an experiment on the given optimal setting validated the model generated during RSM implementation. The second-order polynomial model was applied to predict the response variable (*Y*) as shown below,

(1) $$Y=\beta_{0}+\sum_{j=1}^{4}\beta_{i}X_{i}+\sum_{\text{ij}=1}^{4}\beta_{\text{ij}}X_{i}^{2}+\sum_{i}^{2}\sum_{\text{j}=\text{i}+1}^{4}\beta_{\text{ij}}X_{i}X_{j}$$

where *Y* is the response value (relative activity) and *β*_o_, *β*_i_, *β*_ii_ and *β*_ij_ are the regression coefficients for the interception, linear, quadratic and interaction terms, respectively. *X*_i_ and *X*_j_ were the independent variables.

### 2.3. Characterization of Chitosan

Under the optimal conditions, chitosan was hydrolyzed by pepsin, and the mixed solution after reaction was concentrated to about one-twentieth with a rotary evaporator under reduced pressure. The mixture was neutralized to pH 9.0 and precipitated by adding ethanol. The precipitate was collected after drying over phosphorus pentoxide in a vacuum to get sample LMW chitosan (LMWC) for structural characterization. IR spectral studies were performed in a Perkin Elmer spectrum 2000 spectrometer (CT, Livonia, MI, USA) under dry air at room temperature using KBr pellets. For chitosan and LMWC (2-mg samples in 100 mg of KBr), 20 mg of the mixture were palletized and subject to IR spectroscopy. Scanning electron micrograph (SEM) was obtained with a JEOL JSM-6700F instrument (Tokyo, Japan). For SEM, 0.5-mL aliquots from the above assay tubes were centrifuged in micro-centrifuge tubes. The pellets obtained were treated with phosphate buffer (pH 7.0, 0.3 M), fixed with glutaraldehyde (1%) for 1 h at 4 °C, further treated with 10%–96% alcohol in a sequential manner then dried [3]. The samples (chitosan and LMWCs solids) were spread on a double-sided conducting adhesive tape pasted to a metallic stub, subjected to gold (100 µg) covering and observed at an accelerating voltage of 20 kV. Room temperature UV-VIS spectra of the chitosan and LMWCs solids were recorded on a SHIMADZU UV-2550 spectrophotometer. UV-VIS spectroscopy was performed on the solution of chitosan perchlorate. A solution of chitosan (10^−2^ g/L) was prepared by adding a stoichiometric amount of 10^−1^ M perchloric acid to a calculated dry weight of chitosan in the solid state. Taking into account the water content and degree of deacetylation of the chitosan, it was then stirred to complete dissolution. Weight-average molecular weight (*M*_W_) was measured by GPC. The GPC equipment consisted of connected columns (TSK G5000-PW and TSK G 3000-PW), a TSP P100 pump and an RI 150 refractive index indicator detector. The eluent was 0.2 M CH_3_COOH/0.1 M CH_3_COONa. The eluent and the chitosan sample solution were filtered through 0.45-µm Millipore filters. The flow rate was maintained at 0.1 mL/min. The sample concentration was 0.4 mg/mL, and the standards used to calibrate the column were TOSOH pulman. All data provided by the GPC system were collected and analyzed using the Jiangshen workstation software package. A decrease in the pepsin-catalyzed viscosity of the highly viscous chitosan solution during the two-hour reaction was measured continuously in a Cannon-Fensk (Schott Geraete, Model GMBH—D65719, Mainz, Germany) capillary viscosimeter. The solutions were filtered before determining the viscosity, which was carried out at the lowest shear velocities permitted within the experimental error and the Newtonian plateau. The determination of the depolymerization degree (DD) of chitosan samples was carried out by the linear potentiometric method. This analysis was carried out by dissolving 0.25 g of chitosan in 20 mL of HCl solution, 0.1 N, then filling it up to 100 mL with distilled water. Titration was performed until the chitosan solution reached a pH of approximately 6.5 (range of chitosan non-protonation). Concerning the polydispersity index study, the aqueous solution of sodium alginate (0.1% w/v) was sprayed into the chitosan solution obtained after 1 h and 2 h of hydrolysis (0.1% w/v) containing Pluronic F-68 (0.5% w/v) under continuous magnetic stirring at 1000 rpm for 30 min. Nanoparticles were formed as a result of the interaction between the negative groups of sodium alginate and the positively-charged amino groups of chitosan (ionic gelation). Nanoparticles were collected by centrifugation (REMI high speed, cooling centrifuge, REMI Corp., Mumbai, India) at 18,000 rpm for 30 min at 4 °C. For the particle size and size distribution study, these nanoparticles were redispersed in 5 mL of HPLC grade water. The sample volume used for the analysis was kept constant, i.e., 5 mL to nullify the effect of stray radiations from sample to sample. Studies were carried out in triplicate (*n* = 3), and the standard deviation (SD) was recorded.

## 3. Results and Discussion

### 3.1. Enzymolysis of Chitosan by RSM

#### 3.1.1. Box–Behnken Design Analysis

The hydrolysis yield experiment was conducted using the Box–Behnken design, and the results are presented in Table 2. Statistical analysis of variance (ANOVA) was performed in order to investigate not only the fitness and significance of the model, but also the effects of the individual variables and interaction effects on the response. It was also noticed in this study that the SSR yield (*Y*) was higher within the first 60 min of hydrolysis. This was attributed to the accessibility of the pepsin active sites to the glycosidic bonds of chitosan. According to the ANOVA results (Table 3), the model is highly significant, so it has an important effect on the SSR yield *(Y)* with a *p*-value of less than 0.0001 to predict the response values. In terms of the significant coefficients, the independent variables *p* (initial pH), *T* (reaction temperature) and *E* (pepsin concentration) were highly significant terms (*p* ≤ 0.0001); therefore, pH *P* affects the solubility of chitosan before all other reactants, while pepsin through its concentration *E* under control of temperature *T* proceeds to the hydrolysis of chitosan. In terms of interaction, the terms of *PE*, *TE* and *EC* (*p* < 0.05) were significant terms influencing SSR yield (*Y*). It is quite remarkable that all of the significant interaction terms contain the independent variables *E*. *E* interacts with all other reaction components, implying that the process of hydrolysis does in fact depend on *E*. All of the quadratic terms of the *P*^2^, *T*^2^, *E*^2^ and *C*^2^ were highly significant terms (*p* < 0.0001). The elimination of the insignificant terms could improve the regression model, and the quadratic model was given as:

*Y* = 91.23 + 6.15 *P* − 7.30 *T* + 10.84 *E* − 4.97 *PE* + 6.22 *TE* + 7.19 *EC* − 32.14 *P*^2^ − 28.96 *T*^2^ − 28.60 *E*^2^ − 32.24 *C*^2^ (2)

Equation (2) is in terms of the coded factors.

Equation (2) confirms that those linear and interaction terms were significant in affecting the SSR yield. The positive coefficients (+6.15 and +10.84) of *P* (initial pH) and *E* (pepsin concentration) in Equation (2) signify a linear effect of increasing from *P* 2–4.2 and *E* 50–103.76 mg/L on the SSR yield (*Y*) and then reaching equilibrium when the *P* to *E* further increased. The negative coefficient (−7.30) of *T* (reaction temperature) in Equation (2) indicates a linear effect of decreasing when *T* goes over 50 °C on the SSR yield. Moreover, the interaction coefficient of *PE* in Equation (2) shows a negative effect of decreasing the SSR yield (*Y*), whereas the interaction coefficients of *TE* and *EC* for the equation have a positive effect that increases the yield (*Y*).The determination coefficient (*R*^2^) of the regression model equation was evaluated by the *F*-test for analysis of variance (ANOVA), and the ANOVA statistics for the response *Y* are shown in Table 3. The value of the determination coefficient (*R*^2^ 0.9891) indicated that the quadratic model was statistically significant, advocating for a high correlation between observed and predicted values. The predicted *R*^2^ is a measure of how good the model predicts the values for the response, and the adjusted *R*^2^ verifies the experimental data and model precision. The predicted *R*^2^ and adjusted *R*^2^ were 0.9406 and 0.9782 (both close to one), respectively, which indicates the adequacy of the model and showing that the 0.9406% variability of the response *Y* is capable of explaining the model. The “lack of fit tests” compare residual error to “pure error” from replicated experimental design points. Its *p*-values were greater than 0.05; this response indicated that the lack of fit for the model was insignificant; that is to say, the quadratic model was valid for the present study. Adequate precision measures the signal to noise ratio, and a ratio greater than four is desirable. The adequate precision for *Y* was 30.039, demonstrating an adequate signal. This model can be used to navigate the design space. On the other hand, a relatively lower value of the coefficient of variation (CV 9.39%) indicated the good precision and reliability of the experiments [14]. ANOVA results of these quadratic models indicated that the model can be used to predict the process of chitosan hydrolysis.

#### 3.1.2. Interactions between the Variables

**Figure 1:**
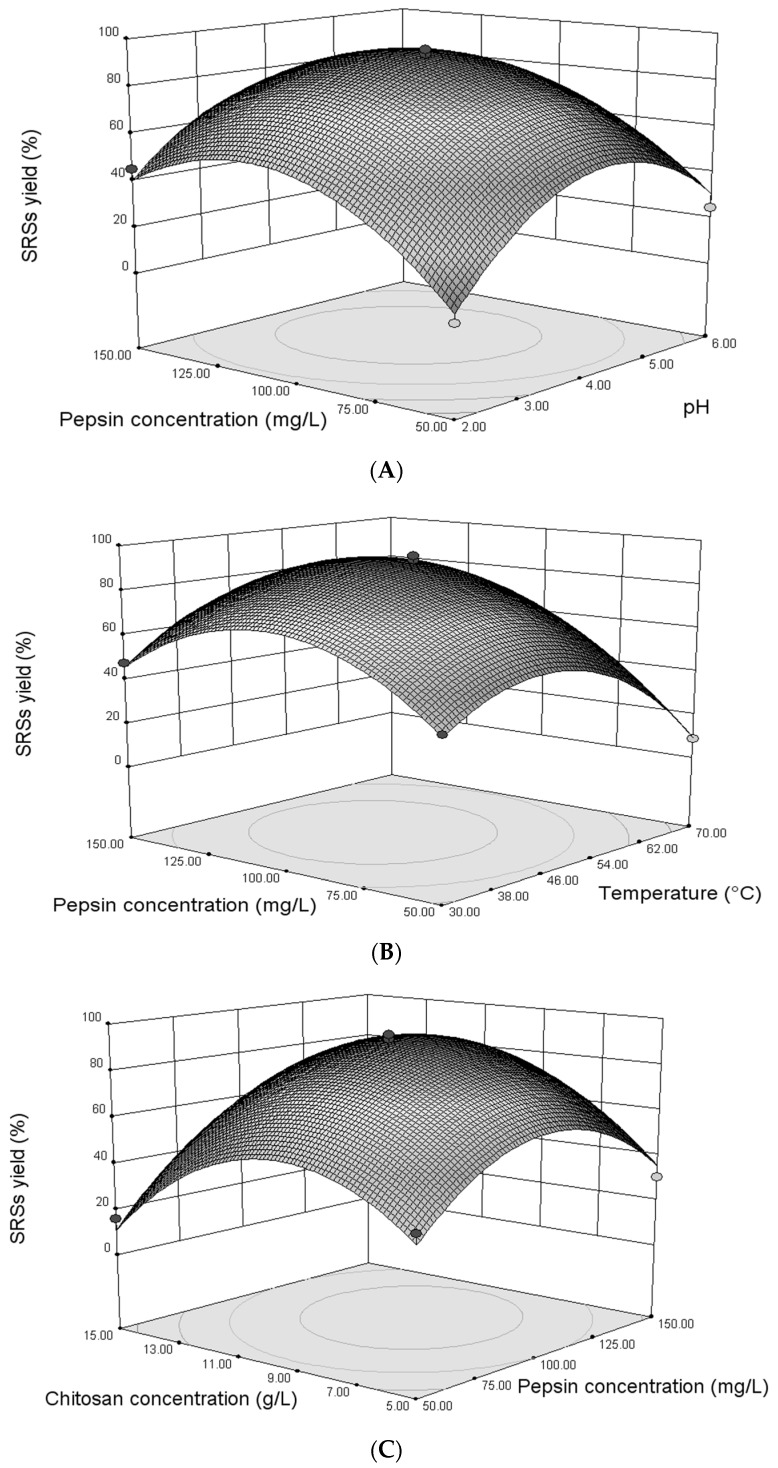
Response surface of predicted reducing sugar (SRS) yield versus pH and pepsin concentration (**A**); reaction temperature and pepsin concentration (**B**); chitosan concentration and pepsin concentration (**C**).

Three-dimensional response surfaces were plotted on the basis of the graphical representations of the regression equation in order to investigate the interaction between the variables, as well as to determine the optimum condition of each factor for maximum enzymolysis for the production of low molecular weight (LMW) chitosan. The model suggested the presence of significant interaction principally between *E* (pepsin concentration) and the three other terms *P* (initial pH), *T* (reaction temperature) and *C* (chitosan concentration). We further characterize the interaction in the range of the process variables. Figure 1A represents the combined effect of *P* and *E* on SRS yield (*Y*), while the other two variables were held at zero. The elliptical nature of the contour plot between *P* and *E* indicates that significant interaction between these two variables had an effect on SRS yield (*Y*). Pepsin concentration *E* demonstrated a quadratic effect on the response, where the SRS yield increased at lower concentrations (<103.76 mg/L), followed by a slight decline with an increase in pepsin concentration. The trend also observed that the SRS yield increased to a maximum with the increase in pH and then gradually decreased to a higher pH (<4.2). It is clear from Figure 1B that the combined effect of reaction temperature *T* and pepsin concentration *E* was significant with the contour curve of an oval shape. The enzyme concentration *E* had almost no direct influence on SRS yield (*Y*); *E* relies rather on the temperature to influence *Y*. However, the temperature demonstrated a quadratic effect on the response, where the SRSs yield (*Y*), increased to the maximum (89.58%) with the increase in temperature and then decreased gradually at a higher temperature (>50 °C). The combined effect of the concentrations of chitosan and pepsin, *C* and *E*, respectively, on the SRS yield (*Y*) is shown in Figure 1C. The contour line with an elliptical shape demonstrates that the combined effect of the chitosan and pepsin concentrations *C* and *E* on the SRS yield (*Y*) is significant. Both concentrations have effects on the SRS yield (*Y*) when the chitosan concentration *C* and pepsin concentration *E* were under 10 g/L and 110 mg/L, respectively, and then achieve a balance with increasing chitosan concentration *C* and pepsin concentration *E*. Pepsin concentration *E* plays a pivotal role in this reaction process. Although the actual situation might be more complicated than what we reported, an attempt for the optimization of enzymolysis was made by RSM.

#### 3.1.3. Optimization Analysis

Canonical analysis is one of the multivariate linear statistical analyses used to locate the stationary point of the response surface and to determine whether it represents a maximum, minimum or saddle point. It is also used to characterize the nature of the stationary points [15]. RSM was used to optimize the desired response of the system, which was the SRS yield (*Y*), and to keep all of the variables in range of the experimental values. The optimal conditions for the production of SRSs by pepsin-catalyzed chitosan enzymolysis by the model equation were as follows: pH 4.16, reaction temperature 47.95 °C, pepsin concentration 108.56 mg/L and chitosan concentration 9.97 g/L. This result differs from the one proposed by Kumar et al., 2007b (pH 5.0 and 45 °C) [6], as well as the one proposed by Tomas. R et al., 2007 (pH 4.5 and 40 °C) [12]. This could be due to the difference in pepsin origin. The theoretical SRS yield predicted under the above conditions was 92.77%. In order to verify the optimization results, experiments with three independent replicates were performed under the predicted optimal conditions. The SRS yield was 91.1% ± 0.4% in optimized conditions of pH 4.0, temperature 50 °C, pepsin concentration 110 mg/L and chitosan concentration 10.0 g/L, which was close to the predicted response and confirmed the efficacy of the predicted model. The results of previous work [13] (Pan et al., 2016) and this one confirm the efficiency of the RSM in the process of optimizing chitosan enzymolysis.

### 3.2. Enzymolysis Kinetic

The hydrolysis kinetics is essential in supplying the basic information for the design and operation of the enzymolysis with the aim of gaining good soluble chitosan products. In order to investigate the enzymolysis kinetics, pepsin was used to degrade chitosan in a solution (pH 4.0) containing initial substrate concentrations ranging from 2.0 to 18.0 g/L with 110 mg/L pepsin at 50 °C for 120 min. The SRS yield in the reaction solution was monitored at regular intervals.

#### 3.2.1. Effect of Chitosan Substrate Concentration

**Figure 2:**
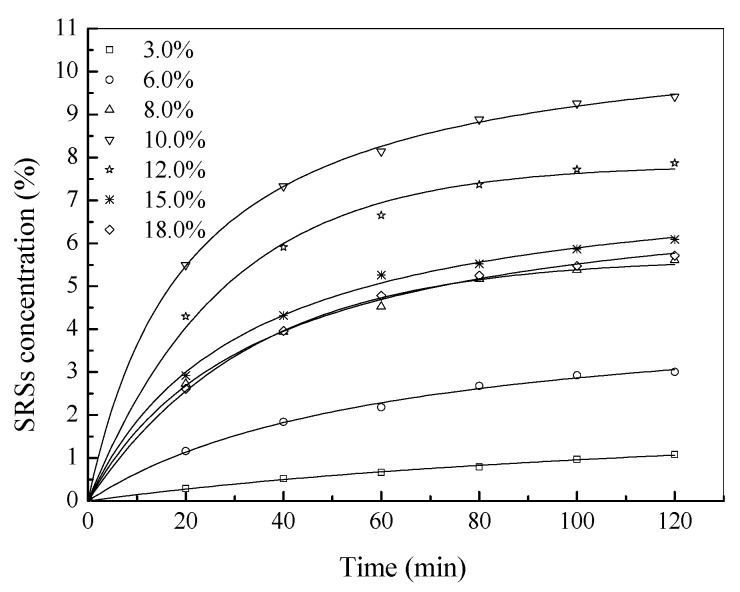
Enzymolysis of chitosan at different substrate concentrations.

The effect of substrate concentration on the catalytic performance of pepsin is shown in Figure 2, which indicates that substrate concentration did not have a significant effect on the equilibrium time. At lower substrate concentrations (2.0~10.0 g/L), the SRS yield increased with substrate concentration. At higher substrate concentrations (>10.0 g/L), the SRS yield decreased with the increase of substrate concentrations. This might be because the viscosity of the reaction system increased with the concentration of chitosan, which further slowed the diffusion of chitosan to the active center of the enzyme molecule resulting in the reduction of enzymatic activity. The results suggest that the chitosan concentration greater than about 10.0 g/L inhibits the activity of pepsin.

#### 3.2.2. Kinetic Constants

For the data obtained in the present study (Figure 2), the kinetic parameters were calculated on the basis of first-order and second-order models [16,17], and the results are shown in Table 4. The results show that all of the correlation coefficients *R*^2^ in the two models are above 0.95, demonstrating their applicability. Comparing the two models, the second-order model (*R*^2^ 0.984~0.989) was more suitable for describing the process of chitosan enzymolysis based on a higher *R*^2^. For the second-order model, the theoretical values (*Q*_e_) were in good agreement with the corresponding experiment values (*Q*_e,exp_). The hydrolysate SRS concentration increased with substrate concentration, and the maximum value *Q*_e_ reached was 10.03 g/L, after which there was a decrease with further increasing substrate concentration. A similar trend is also found in the hydrolysis rate constant *k*_2_, demonstrating that chitosan had an inhibitory effect on pepsin activity at high concentrations (> about 10.0 g/L). In the second-order model, the constant *k*_2_ was the specific hydrolysis rate (*v*) and was used to calculate the initial hydrolysis rate *h*, at *t*→0 [18], as follows.

*h* = *k*_2_*Q*_e_^2^ (3)

where *Q*_e_ is the SRS concentration in the reaction solution at equilibrium.

The initial hydrolysis rate *h* increased with substrate concentration from 3.0 to 10.0 g/L and then decreased due to the inhibitory effect of the chitosan as its concentration was further increased. The enzymolysis process in the presence of the inhibition of a substrate to the enzyme could be described by the Haldane model, and the Haldane equation is presented by Equation (4) [19,20],

(4) $$v=\frac{V_{\text{max}}S}{K_{m}+S+\frac{S^{2}}{K_{\text{ss}}}}$$

where *v* is the specific rate of hydrolysis, which is equal to the hydrolysis rate constant *k*_2_. *K*_ss_ is the inhibition constant. *V*_max_ is the maximum rate of hydrolysis. *K*_m_ is the Michaelis rate hydrolysis constant. *S* is the substrate concentration.

**Figure 3:**
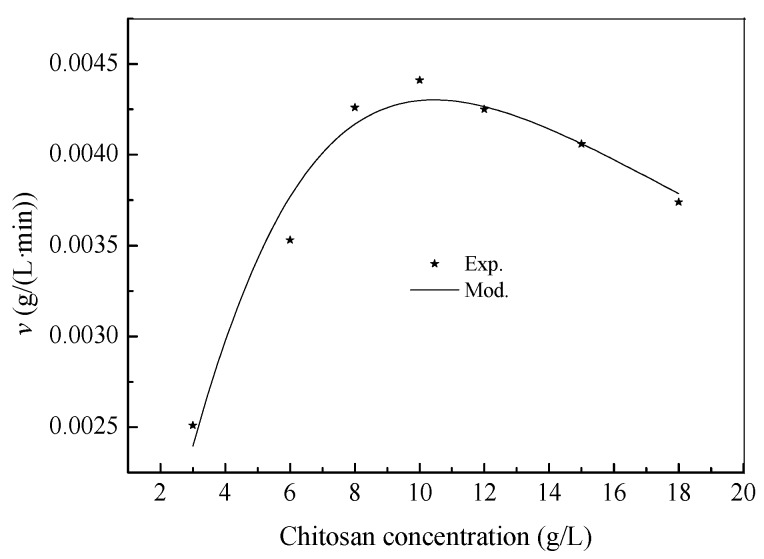
Maximum specific hydrolysis rate for chitosan substrate concentrations from 3.0 to 18.0 g/L.

The relationship between the specific hydrolysis rate (*v*) and chitosan concentrations is shown in Figure 3, where the experimental *v* sharply increased when chitosan concentrations were lower than 10.0 g/L, whereas the inhibition effect of chitosan gradually became prominent at above 10.0 g/L. The value of *R*^2^ is 0.9463, which suggests that the Haldane model had excellent fits to the experimental data. Using a nonlinear least squares regression analysis of Origin 9.0, the kinetic parameters of the experimental data in Figure 2 were determined as follows: *V*_max_ = 0.0043 g/(L·min), *K*_m_ = 46.6244 g/L and *K*_ss_ = 2.3395 g/L. It can be noted that the substrate had been inhibitory, making it impossible to observe an actual *V*_max_. Therefore, *K*_m_ takes on a hypothetical meaning. When *d_v_*/*d*_S_ equals zero, Equation (5) will go through a maximum value at substrate concentration *S*^∗^ and specific hydrolysis rate *v*^∗^. The values can be calculated as follows [21],

(5) $$S^{*}=\sqrt{K_{m}K_{\text{ss}}}$$

(6) $$v^{*}=\frac{V_{\text{max}}}{1+2\sqrt{K_{m}/K_{\text{ss}}}}$$

Equation (6) reflects that the hydrolysis potential of pepsin was determined by the *K*_m_/*K*_ss_ ratio, not just by *K*_ss_ alone. The larger the *K*_m_/*K*_ss_ was, the lower the hydrolysis potential was. According to these values (*V*_max_, *K*_m_ and *K*_ss_), *S*^∗^ and *v*^∗^ were computed as 10.4440 g/L and 0.0002 g/(L·min), and the minimum chitosan inhibitive concentration was 10.44 g/L. Below this concentration, hydrolysis seemed to be increasingly suboptimal, and above this concentration, hydrolysis was inhibited increasingly due to substrate inhibition. At high chitosan concentrations, the enhancement of the viscosity in the reaction system increased the hindrance to diffusion of chitosan molecules. It also restrained the diffusion between the product and activity sites of pepsin, leading to decreased catalytic activity. The calculated results suggested that in order to avoid substrate inhibition, the process of enzymolysis should be operated at a chitosan concentration below 10.5 g/L, which is close to the experimental value of 10.5 g/L. These results could be significant towards understanding the capacities of pepsin for chitosan degradation. Considering the results from two different works on the subject (Pan et al., 2007 [13], and this one), the process of chitosan enzymolysis seems to generally follow the pseudo-second-order and Haldane models.

### 3.3. The Structural Properties of the LMW Chitosan

#### 3.3.1. FTIR Analysis

**Figure 4:**
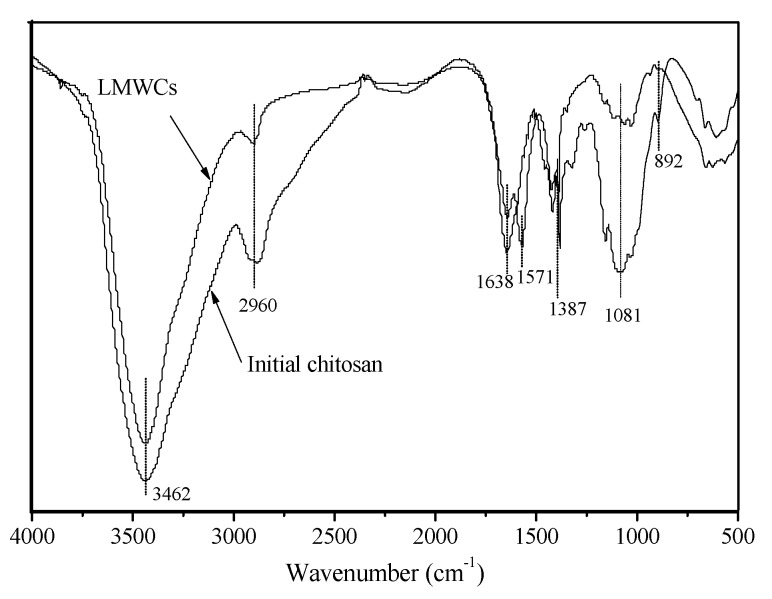
FTIR spectra of the initial chitosan and low molecular weight chitosan (LMWC) samples.

Figure 4 shows the FTIR spectra of the initial chitosan and degraded chitosan LMWC samples where the initial chitosan and LMWCs show basically similar FTIR spectra. The peaks at around 3462, 2960, 1638, 1387, 1081 and 892 cm^−1^ represent the presence of the –OH group, the –CH_2_– group (aliphatic group), the –C=O group, the C–O stretching of the primary alcoholic group (–CH_2_-OH is considered to be a potential site for cross-linking), the –OH group and the β (1→4) glucoside bond in chitosan, respectively [5,22]. These results demonstrated that the structures of the main chain of the initial chitosan and LMWCs were the same. The NH_2_ amino groups had a characteristic peak near 3462 cm^−1^, which was overlapping by the peak due to the –OH group [23]. The occurrence of absorption peaks at around 2900 cm^−1^ was assigned to the asymmetric stretching vibration of the –CH_2_–, and the rapid reduction in the intensity for the LMWCs was probably attributed to degradation of chitosan after hydrolysis. In contrast with the initial chitosan, a significant new peak at 1571 cm^−1^ in the LMWCs, the N–H bending mode of –NH_2,_ was noticeably split at around 1638 cm^−1^, which suggests that the –C=O groups had more opportunity to form stronger hydrogen bonds, and the scission of polymer chains led to the decrease of the chitosan molecular weight [8]. The results indicated that there was no significant difference between the main structures of the two samples before and after the enzymatic hydrolysis, but the molecular weight of the main hydrolysis products decreased, which was in good agreement with Kumar et al., 2007b [6].

#### 3.3.2. UV-VIS Analysis

**Figure 5:**
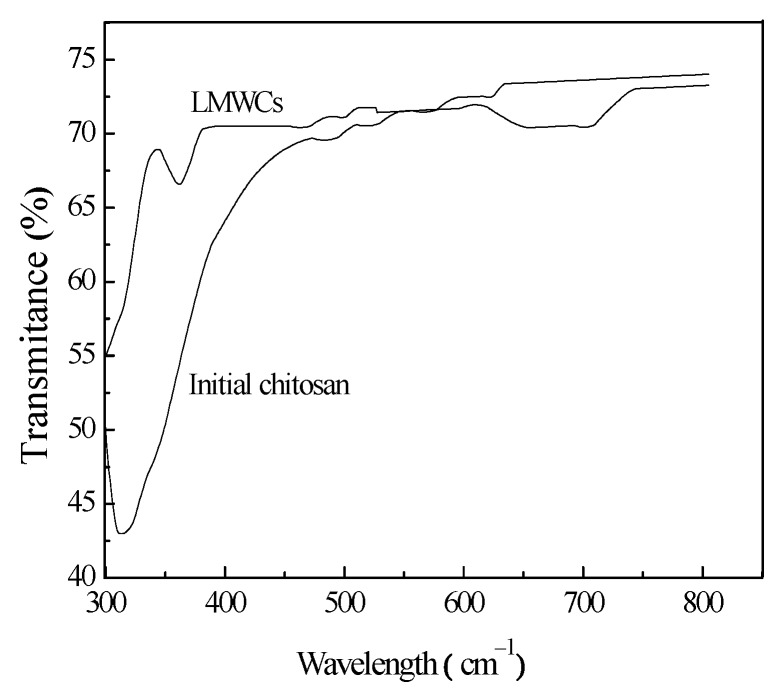
UV-VIS transmittance spectra of initial chitosan and LMWC samples.

Figure 5 shows the optical transmittance spectra for the initial chitosan and LMWCs in the range of 300–800 cm^−1^. The two samples showed very good transmittance in the visible region. For the initial chitosan, low transmission intensity can be observed below the 317-cm^−1^ wavelength; therefore, the transmission intensity started to increase at 317 cm^−1^, until it reached 70.16% at 463 nm. The transmittance of the LMWCs immediately started to increase at 300 cm^−1^, up to 70.80% at 380 cm^−1^. A new absorption peak not appearing in the initial chitosan sample was observed at around 344 cm^−1^, which suggested the existence of a chemical reaction in the LMWCs. The peak could be assigned to the n→π* transition for the carboxyl group in the LMWCs formed after the main chain scission of chitosan [23]. The result of FTIR analysis further confirmed that carbon-oxygen double bonds formed after the degradation of chitosan occurred by the ring opening, turning chitosan into one with low molecular weight.

#### 3.4.3. SEM Analysis

**Figure 6:**
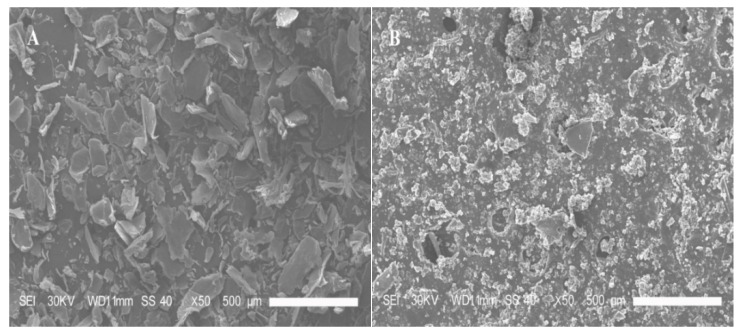
SEM images of the initial chitosan (**A**) and LMWC (**B**) samples, ×50.

The surface and internal structure of the LMWCs were evaluated using SEM, and the results are shown in Figure 6. The initial chitosan presented a heterogeneous structure, which consisted of random-sized, loose particles with irregular edges. For the LMWCs, the loose granular nature of chitosan was observed after enzymolysis, which seemed to have taken place homogeneously in bulk. The enzymolysis made the surface of the hydrolyzed chitosan denser and more porous, leading to a change in the surface morphology, thus exhibiting a thick, dense, but porous structure with small cavities distributed on the entire surface of the LMWCs. The discrepancies in the observed microstructure between the initial chitosan and LMWCs might also be attributed to their different inter- or intra-particle hydrogen-bonding systems. It was further confirmed by SEM analysis that chitosan was degraded into small molecular weight chitosan (LMWC) when the chain of chitosan macromolecules was broken into smaller unit.

#### 3.4.4. Depolymerization Degree, Viscosity, Molecular Weight and Polydispersity Index Analysis

The DD, *M*_W_, average viscosity and polydispersity index of chitosan and degraded chitosans (LMWCs and chitosan oligosaccharides (COS)) are listed in Table 5. The DD of chitosan increased with the decrease in molecular weight and viscosity. The average viscosity and molecular weight value of chitosan decreased by approximately 86% in relation to the molecular weight value during the first 60 min of reaction [6]. In the presence of the optimal conditions of hydrolysis, the polysaccharide chains are submitted to degradation due to the efficiency of the different parameter and the prolonged times necessary for obtaining advanced depolymerization. The polydispersity index (PDI = *M*_W_/*M*_n_) was studied, and after 2 h of hydrolysis, there was no trace of monomers; contrary to our previous study with papain [13], where trace levels of monomers were detected. There were only chitosan oligosaccharides (COS) and low molecular weight chitosan (LMWC) in different proportions according to the time of hydrolysis (Table 5). These could be interpreted as confirming that chitosan was degraded into smaller molecular weight units.

## 4. Conclusions

The high molecular weight chitosan shows poor solubility in aqueous solutions, and the high viscosity of its solution limits its applications. To improve its solubility, as well as biological, chemical and physical properties, enzymolysis by pepsin was employed to prepare low molecular chitosan. RSM was launched to investigate the influence of process variables on the DH followed by a BBD approach. The optimized conditions were a 10.0-g/L chitosan concentration of pH 4.0 at 50 °C, a 110-mg/L pepsin concentration and enzymolysis time of 70 min, where the predicted value of the DH was 91.1%.

The enzymolysis process of chitosan follows the pseudo-second order and Haldane models, wherein for more than a 10.5-g/L concentration chitosan, pepsin undergoes severe inhibition due to the viscosity increase, causing an increase in the barrier released in the reaction system. Based on the characteristic analyses by FTIR, UV-VIS and SEM, hydrolyzed product LMWCs almost retained the backbone of the chitosan macromolecular structure. The breaking of the C-O-C glycosidic bond led to chain scission and the formation of carbonyl groups. Therefore, the degradation method was feasible, convenient and potentially applicable.

## Figures and Tables

**Table 1:** Experimental range and levels of the independent variables.

Independent Variables	Symbols	Units	Code Levels		
			−1	0	1
pH	*P*		2	4	6
Temperature	*T*	°C	30	50	70
Enzyme concentration	*E*	mg/L	50	100	150
Chitosan concentration	*C*	g/L	5.0	10.0	15.0

**Table 2:** Experimental Box–Behnken design matrix and its response and predicted value.

Run	Experimental Variables				Response *Y* (%)	
	*P*	*T* (°C)	*E* (mg/L)	C (g/L)	Expt.	Predicted
1	6.00	30.00	100.00	10.00	39.76	40.092
2	4.00	50.00	100.00	10.00	89.58	91.232
3	6.00	50.00	100.00	15.00	30.06	27.680
4	2.00	70.00	100.00	10.00	13.52	13.202
5	4.00	70.00	150.00	10.00	43.74	43.440
6	4.00	50.00	50.00	5.00	32.58	28.142
7	2.00	50.00	50.00	10.00	4.950	8.538
8	4.00	50.00	100.00	10.00	92.28	91.232
9	6.00	50.00	100.00	5.00	40.23	38.320
10	6.00	50.00	150.00	10.00	44.78	42.515
11	4.00	30.00	150.00	10.00	47.58	45.598
12	2.00	50.00	150.00	10.00	45.02	40.158
13	4.00	50.00	100.00	10.00	89.94	91.232
14	4.00	50.00	150.00	5.00	30.48	35.437
15	4.00	50.00	50.00	15.00	15.91	10.967
16	6.00	50.00	50.00	10.00	24.59	30.775
17	4.00	50.00	100.00	10.00	90.14	91.232
18	4.00	70.00	50.00	10.00	8.680	9.325
19	6.00	70.00	100.00	10.00	32.44	32.478
20	4.00	50.00	150.00	15.00	42.58	47.032
21	4.00	70.00	100.00	5.00	25.49	24.970
22	4.00	30.00	100.00	15.00	34.93	36.773
23	2.00	50.00	100.00	5.00	17.13	18.173
24	4.00	70.00	100.00	15.00	20.05	20.505
25	4.00	30.00	50.00	10.00	37.39	36.353
26	2.00	30.00	100.00	10.00	34.80	34.775
27	4.00	50.00	100.00	10.00	94.22	91.232
28	2.00	50.00	100.00	15.00	22.66	23.233
29	4.00	30.00	100.00	5.00	37.02	37.888

**Table 3:** ANOVA analysis for the response surface quadratic model (*α* = 0.05)

Source	Sum of Squares	DF	Mean Square	*F*	*p*-Value
Model	18,573.62	14	1326.69	90.55	<0.0001
P	453.62	1	453.62	30.96	<0.0001
T	638.90	1	638.90	43.61	<0.0001
E	1410.07	1	1410.07	96.24	<0.0001
C	23.35	1	23.35	1.59	0.2274
PT	48.72	1	48.72	3.33	0.0896
PE	98.80	1	98.80	6.74	0.0211
PC	61.62	1	61.62	4.21	0.0595
TE	154.63	1	154.63	10.55	0.0058
TC	2.81	1	2.81	0.19	0.6683
EC	206.93	1	206.93	14.12	0.0021
P 2	6699.96	1	6699.96	457.30	<0.0001
T2	5438.75	1	5438.75	371.22	<0.0001
E2	5304.36	1	5304.36	362.05	<0.0001
C2	6742.76	1	6742.76	460.22	<0.0001
Residual	205.12	14	14.65		
Lack of Fit	189.50	10	18.95	4.85	0.0708
Pure Error	15.62	4	3.90		
Cor Total	18,778.73	28			
*R*^2^	0.9891				
Adjusted *R*^2^	0.9782				
Predicted *R*^2^	0.9406				
Adeq precision	30.039				
CV	9.39				

**Table 4:** Kinetic parameters for the chitosan enzymolysis by pepsin at 50 °C.

Chitosan Concentration (g/L)	First-Order *Q*_t_ = *Q*_e_(1 − exp(−*k*_1_*t*)) *		Second-Order *t/Q*_t_ *=* 1/(*k*_2_*Q*_e_^2^) *+* *t*/*Q*_e_ *		
	*k*_1_ (1/min)	*R*^2^	*k*_2_ (L/(g·min))	*H* (g/(L·min))	*R*^2^
2.0	0.00946	0.99543	0.00251	2.48935	0.99632
6.0	0.01966	0.99522	0.00353	4.62264	0.99618
8.0	0.0301	0.99593	0.00426	7.15633	0.99904
10.0	0.04086	0.99465	0.00441	11.07718	0.99954
12.0	0.03626	0.99513	0.00425	9.54351	0.99935
15.0	0.03109	0.99857	0.00406	7.76309	0.99813
18.0	0.02874	0.99956	0.00374	7.48846	0.99882

* *Q*_t_ and *Q*_e_ are the SRS concentration at *t* and equilibrium, respectively; *k*_1_ and *k*_2_ are the rate constants of the first-order and second-order models, respectively.

**Table 5:** Properties of degraded chitosan.

Source	*M*_w_ (×10^3^)	DD (%)	Viscosity Decrease (%)	Yield (%)
Native	300	-	-	-
CH_1_	195.4	74.60	86	-
CH_2_	65.9	92.00	93	-
Monomers ^2^	-	-	-	n.t.
LMWC ^1^	25–20	-	-	28.26
LMWC ^2^	13–9	-	-	35.04
COS ^1^	90–85	-	-	71.74
COS ^2^	65–50	-	-	64.94

^1^: after 1 h; ^2^: after 2 h; CH_1_: chitosan hydrolyzed after 1 h; CH_2_: chitosan hydrolyzed after 2 h; monomers: sum of GlcN and GlcNAc; COS: chitosan oligosaccharides; LMWC: low molecular weight chitosan; n.t.: no trace.
